# Report of the Physician of the Erie County Poorhouse

**Published:** 1856-11

**Authors:** 


*Report of the Physician of the Erie County Poorhouse.*—The Annual Reports of our public institutions are fruitful themes for the public journalist. The report of J. D. Hill, for the year ending October, 1856, is in the usual scattering style of that personage. Its syntax and prosody might be mended Such sentences as the following rather puzzle the youthful student of parsing:

"Purulent Opthalmia which had been so long the *pestilence* of the children's department and only the previous year, over one hundred and twenty suffered from its blighting influence, has been, and is now, with the rare exception of a few sporadic cases, entirely free from its baneful and loathesome presence."

The casual reader might inquire from what baneful presence it is, that purulent ophthalmia is free? or infer that the sporadic cases were not so fortunate as the others, or were compelled to submit to some uncomfortable companionship. We should not, however, expect too much from poor-house literature.

To go into the merits of the report. The Dr. announces that "so far as consistent with the facilities and *other arrangements* of the Institution," he has classified the sick. He then proceeds to give the usual disinterested complimentary notice to his employers, the Superintendents. The House Student is a paragon, and we cannot sufficiently admire the English, *a la* Malaprop and Partington, of this, his brief, but double-distilled eulogy.

"Of the zeal, prudence and untiring efforts of the House Student, I cannot speak in terms of praise beyond merit. The conscientious regard to the duties of place, the laudable union of responsibility, with the benevolence and charity evidenced in unremitted devotion and anxious care for the sick; exceeds, while it deserves the highest commendation I could pronounce."

Edward Everett is no longer the prince of eulogists.

Come we now to statistics. The Dr. having placed his student among the *Dii Minores*, proceeds to his own apotheosis. We quote in full, allowing him to speak for himself, for surely never man spake as this man:

"The following are (It are, are it?) the number of deaths in each, (each what?) for the ten past years:

| Year. | Mortality. | Year. | Mortality. |
|---|---|---|---|
| 1847. | 113 | 1852. | 172 |
| 1848. | 159 | 1853. | 143 |
| 1849. | 158 | 1854. | 170 |
| 1850. | 155 | 1855. | 82 |
| 1851. | 114 | 1856. | 55 |

| | |
|---|---|
| Deduct from the reported mortality for the past year: | |
| Still-born, | 2 |
| Brought to the alms-house in a dying condition, | 9 |
| This leaves the mortality of the inmates, | 44 |
| Deduct from this 44, those cases which when admitted were incurable, and must necessarily terminate in death: | |
| Of Consumption, | 13 |
| Malignant Cancer, | 2 |
| Organic Disease of the Brain, | 2 |
| Sudden and unavoidable death of the two infants not treated, | 2 |
| This would leave the mortality of the inmates who were appropriate subjects for medical treatment, | 35 |

which mortality I do not hesitate will compare favorably with any institution of like character in this county.

"I think it not improper or invidious to state that I believe the mortality the first year which I had the charge would have been less, if not as small as the last, had it not been for uncontrollable circumstances connected with the condition of the sick and the ill-arrangements of the hospital department at the time it came under my care.

"I have given the above mortality for the past ten years, for the purpose of showing that the Alms House in its management, is not, what it has been maliciously represented to be by a mercenary clique, since my connection with it. But I claim, as now conducted, it is a beneficent institution, directed by intelligence on the liberal principles of human kindness and christian philanthropy. That it is the tax-paying citizens who contribute willingly and liberally by assessment for its support, designed it should be—an asylum for the helpless orphan and destitute—a retreat where the indigent sick and unfortunate may be the recipients of judicious care and competent treatment."

It is necessary, to the proper understanding of this marked reduction in the mortality of the Erie County Poor-House, to state that, during the two years past, great reforms have been introduced into its construction and its entire sanitary arrangement. To this, and to the succession of two healthy seasons, is due, as we believe, the diminished mortality of the Poor-House. The history of that reform constitutes one of the most interesting features in the history of the Buffalo Medical Journal. It will flatter not only our own personal vanity, but that of our predecessor in the editorial chair, to refer to this history; and it will at the same time show how much may be accomplished by an intelligent censorship on the part of the public press.

We use no harsh language in saying that, at various periods in the history of its management, the Erie County Poor-House has been a disgrace to the county, and a byeword among philanthropic men. Going back to the number of this Journal for December, 1848, we find the following comments from the pen of Prof. Flint. The number of persons relieved for the preceding year had been 984; the number of deaths had been 159: giving a ratio of mortality of one out of every six!

Says Prof. Flint—"This enormous mortality is a melancholy commentary on the *relief* afforded to the sick poor of the county of Erie, at the poor-house. We hope, for the honor of humanity, that a parallel cannot be found in the poor annals of this country. * * * * * * The mortality is nearly twice as large at the Erie county poor-house as at the Charity Hospital, at New Orleans. * * * * * * The mortality of the Erie county alms house is thus nearly four times greater than at the Bellevue Hospital," &c., &c.

The next year, 1849, the mortality was decreased so that *only* one out of every eight died. After reasoning from the facts given, for a little, Prof. Flint says: "The inflexibility of arithmetic leaves no room for incredulity. A truth so lamentable should not be concealed. Let it be sounded in the ears of community, that, of the sick persons whom public charity assumed to provide for, at the poor-house, during the past year, at least *sixty-two* have been sacrificed to the want of needful hospital advantages for recovery."

We regret that our space will not allow us to quote more largely from that succession of noble defences of the cause of humanity, which at that time emanated from the pen of our predecessor in the management of this Journal. They had their effect. Denunciation followed denunciation, until the poor-house, pest-house, plague spot, on Prospect Hill, was deserted, and a new edifice erected some five miles out of the city. Something was gained, but the stubborn ignorance of the authorities contrived, almost in the face of impossibilities, to make the new house as murderous as the old. Year by year the mortality went on increasing instead of diminishing. In the summer of 1854, the mortality was so appalling that the present editor took up the war, in the absence of Prof. Flint, and proved that the new house was as great a disgrace as the old. First in the *Commercial Advertiser*, of this city, and next in this Journal, we attacked its policy again and again. In October, 1854, we published a series of articles, from one of which we extract the following:

"In New York Hospital the mortality is 1 in 11$\frac{1}{8}$. In Charity Hospital, New Orleans, it is 1 in 11. In Pennsylvania Hospital, it is 1 in 11$\frac{87}{100}$. In Quarantine Hospital, (N. Y.) during the ship-fever of 1847, it was 1 in 16. In Erie County Poor-House it is 1 in 9$\frac{16}{100}$. Is it possible that a more fatal class of cases reaches our poor-house than enters the hospitals of our largest cities? Let it be recollected that this is the ratio of deaths in a house which receives lunatics, idiots, cripples, and destitute poor in much larger proportion than the sick proper, and that a large proportion of its inmates remain but a short time."

Such was the condition of the Erie county poor-house from 1849 to 1854, inclusive. The repeated attacks made upon this murderous policy emanated —every one of them, whether published in our own columns or in the daily papers—from one or the other of the two editors of this Journal. The wide publicity thus given to the discussion, drove the Board of Supervisors into an unwilling reform. No one was louder in denouncing our motives, no one denied more vigorously the necessity for reform, than the person who now holds the position of Poor-House Physician; and brazenly claims the diminished mortality as the result of his professional skill! The claim is nauseating.

Well. The Board of Supervisors commenced a miserably insufficient reform of the house. Providence interfered and burned down the building just at the nick of time. A majority of the superintendents acknowledged their duty, and strove through much opposition to perform it. The result was a house, not excellent, but very well constructed, with due reference to sanitary laws. And as the direct and positive result of that change in construction, we have this diminished mortality. The reform began, continued, and ended in this Journal. Without this Journal, Dr. Hill would now be having half as many deaths in the house, per annum, as he earns dollars for attending it; as it is, he gets nearly eight dollars per death.

Our only reward, the only recompense we ask in return for the persecution and pecuniary loss we have suffered in this controversy, is the proud reflection that our labors have not been fruitless, that we have accomplished our object in the main. The lives and interests of the poor are measurably protected, we have no longer an annual hecatomb of victims offered up on the shrine of a beggarly economy. And we hope to see a day when *our* policy shall be confided to the hands of men whose hearts have some sympathy with humanity.

One word more in reference to this unequalled report. The deductions made from the list of deaths seem to prove, (*vide* the figures!) that the 35 cases "who were appropriate subjects for medical treatment," died unnecessarily. This is the logical inference, though, doubtless, not the one intended. Again, the "mercenary clique" alluded to, includes some sixty-five out of seventy regular physicians in the city of Buffalo. The grammatical construction of the whole report is such as to make us proud of our membership in a "learned profession."

As a final morceau, we may add that the report claims that more than half the recent cases of insanity admitted have been cured. Among these recent cases are included eighteen patients with delirium tremens!!

